# Stomach Digestion in the Horse

**Published:** 1883-01

**Authors:** 


STOMACH DIGESTION IN THE HORSE.—The digestion of the horse is a matter of especial interest to veterinarians since so many of the horse's ailments result from disturbances in some part of the alimentary tract. We take great pleasure, therefore, in presenting to the profession the results of one of the most careful studies upon this point that has ever been made. Ellenberger and Hofmeistir, in a series of articles in the *Archiv für Wissenschaftliche und Practische Thierheilkunde* report the results of numerous experimental researches regarding stomach disgestion in the horse. The following is a resume of their work:

1. Stomach digestion in the horse is of more importance than had heretofore been supposed.

2. Stomach digestion continues from one meal time to another. There is always food in the horse's stomach when he begins a meal. If food is kept from him, stomach digestion is very slow and the stomach is not empty for at least twenty-four hours.

3. The stomach contents after feeding with oats is a relatively dry, crumbling mass, containing 60 to 70 per cent. of water. After a meal of hay, it is more moist and contains 75-80 per cent of water.

4. The reaction of this mess is always acid.

5. The amount of acid in the gastric juice rarely exceeds 2 per cent. Directly after feeding it is least in amount, being only about .08 per cent. It then gradually increases.

6. The gastric juice of the horse is less acid than that of carnivora.

7. The acid is at the beginning of digestion organic, *i. e.*, it is lactic acid. Later, hydrochloric acid appears, but lactic acid is always present.

8. A feed of oats makes the gastric juice more acid than does hay.

9. Inorganic acids check the transformation of starch into sugar when present in proportion of .02 per cent. Organic acids do not check it until it reaches the ratio of .04 per cent. Hence in the early part of digestion some sugar may be formed in the horse's stomach.

10. In the horse's stomach there was found *(a)* a proteolytic ferment (pepsin proper), *(b)* an amylolytic ferment (diastase of saliva), *(c)* a lactic acid ferment.

11. The turning of starch into sugar is most active in the first one or two hours of digestion. It gradually diminishes and ceases after five or six hours. The larger a meal the longer the sugar-making process continues, because more saliva is swallowed.

12. The amount of sugar in a horse's stomach at the beginning of digestion is, after feeding with oats, 2 per cent., later it is 1 per cent. The amount then decreases. The total amount on the average is at first ʒ i. — ʒ i. $\frac{1}{4}$; later ℥—. After hay feeding the amount of sugar found is less, averaging 3 per cent., or ʒ i. $\frac{1}{4}$ ʒ ii.

13. The digestion of vegetable albumen takes place actively in the stomach. This albumen is turned into peptone. In the early stages the process is very slow. After a moderate meal it reaches its height in three to four hours; after a full meal in six to eight hours. It follows that heavy meals should not succeed each other sooner than from six to eight hours.

