# Erratum

**Published:** 1883-01

**Authors:** 


### Progress of Veterinary Science.

*Erratum*—On page 54, 11th line, after 3 insert ½.

